# PET Imaging in Animal Models of Alzheimer’s Disease

**DOI:** 10.3389/fnins.2022.872509

**Published:** 2022-05-24

**Authors:** Baosheng Chen, Bernadette Marquez-Nostra, Erika Belitzky, Takuya Toyonaga, Jie Tong, Yiyun Huang, Zhengxin Cai

**Affiliations:** PET Center, Radiology, Yale School of Medicine, New Haven, CT, United States

**Keywords:** positron emission tomography, Alzheimer’s disease, β-amyloid (Aβ), tau, neurodegeneration, SV2A, neuroinflamamation, animal model

## Abstract

The successful development and translation of PET imaging agents targeting β-amyloid plaques and hyperphosphorylated tau tangles have allowed for *in vivo* detection of these hallmarks of Alzheimer’s disease (AD) antemortem. Amyloid and tau PET have been incorporated into the A/T/N scheme for AD characterization and have become an integral part of ongoing clinical trials to screen patients for enrollment, prove drug action mechanisms, and monitor therapeutic effects. Meanwhile, preclinical PET imaging in animal models of AD can provide supportive information for mechanistic studies. With the recent advancement of gene editing technologies and AD animal model development, preclinical PET imaging in AD models will further facilitate our understanding of AD pathogenesis/progression and the development of novel treatments. In this study, we review the current state-of-the-art in preclinical PET imaging using animal models of AD and suggest future research directions.

## Introduction

Dementia is a category of neurodegenerative diseases that mainly affect the daily lives of older people and is characterized by progressive loss of memory, communication, problem-solving/thinking, and motorsensory abilities. The common types of dementia include vascular dementia, frontotemporal dementia, dementia with Lewy bodies, and Alzheimer’s disease (AD), which is the most common type and accounts for 60–80% of overall dementia cases (Alzheimer’s Association, 2021). Globally, there are 350,000 new cases of early onset dementia per year, and by 2050, 107 million people are predicted to be living with AD, among which 68% reside in the low- and middle-income countries (Global Burden of Disease Study).

The pathological hallmarks of AD are β-amyloid (Aβ)-containing extracellular plaques and oligomers and tau-containing intracellular neurofibrillary tangles (NFTs). The plaques and oligomers interfere with neuron-to-neuron communication at synapses, leading to neurodegeneration. Tau tangles block the transport of nutrients and other molecules inside the neurons, which contributes to neural death. In addition, the Aβ plaque and tau proteins can activate the microglia, which clears these toxic proteins and dead cells but may result in chronic inflammation (Long and Holtzman, 2019). Atrophy, a decrease in brain volume owing to the loss of synapses, dendrites, and neuronal cell bodies, is another biomarker for AD progression (Pini et al., 2016; Halliday, 2017). In addition, the decrease in glucose metabolism further compromises the brain’s function (Wang et al., 2016). Familial early-onset AD (FAD) is associated with mutated genes such as APP, PSEN1, PSEN2, and MAPT, which also significantly increase the risk for late-onset AD (LOAD) (Ryan and Rossor, 2010), while apolipoprotein E variant ε4 (APOEε4) (Kim et al., 2009) and triggering receptor expressed on myeloid cells 2 (TREM2) are associated with the highest risk of developing LOAD (Wolfe et al., 2018). These dominantly inherited Alzheimer’s disease (DIAD) caused by rare genetic mutations are associated with increased levels of Aβ and tau, decreased glucose metabolism, and brain atrophy 10–20 years before the symptoms set in. In addition, multiple enzymes are associated with AD, including the β-site APP cleaving enzyme 1 (BACE1) (Das and Yan, 2017), caspase 3 (Rohn, 2010), and aspartyl cathepsin (Haque et al., 2008), among others.

**FIGURE 1:**
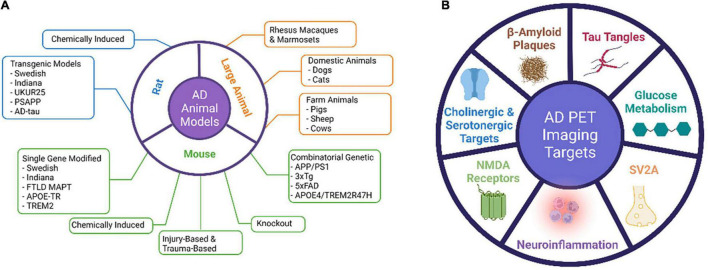
Major AD animal models and PET imaging targets.

Animal models of AD have become essential for studying the pathogenesis and progression of AD pathology and for validating the mechanism of action of novel therapeutics before translation to human trials (Alzheimer’s Association, 2021). Many animal models have been developed to mimic the pathophysiological processes and progression mechanisms of AD and to preclinically test treatment methods (Figure 1). Both invertebrate and vertebrate animals have been used in modeling the aging and certain aspects of AD processes, as genetically modified animals that recapitulate certain traits of AD are needed to understand the pathological and biological mechanisms of AD. Because there has not been a rodent model that completely recapitulates human AD, rodent models have been mainly used for proving mechanisms of action for therapeutic interventions or testing the target binding specificity of imaging probes. A fully characterized AD animal model with stable phenotypes and a clear disease onset time can greatly help address the specific scientific questions.

Many neuroimaging methods, such as magnetic resonance imaging (MRI; structural and functional), computerized tomography (CT), and positron emission tomography (PET), have been increasingly employed to evaluate AD neurodegeneration. PET imaging uses radiolabeled tracers to detect and quantify cerebral and metabolic changes by targeting specific biomarkers that are associated with AD. Fluorodeoxyglucose (FDG) PET detects brain metabolism and amyloid PET that quantifies the amyloid deposit has been developed to understand AD pathogenesis and to monitor disease progression and therapeutic effects. Other neuro-specific, inflammation- and metabolic-associated radiotracers are under development for AD studies. In this study, we discuss the AD animal models that are relevant in AD PET imaging studies and summarize the PET tracers that have been tested in AD animal models and the findings from these studies.

## AD Animal Models

### Mouse Models

In the field of neurodegenerative diseases, mice are the most commonly used animals for their biological features that are similar to those of humans, easily manipulated genetics to mimic human conditions and diseases, and a relatively short life span (1.5–2 years). Because mice do not develop AD naturally, transgenic mice are generated to recapitulate certain AD pathological features to fit the research needs (Table 1). The genes associated with the early onset of AD have been the main targets for transgenic manipulations.

**TABLE 1:** List of major AD mice models.

#	Strain name	Genetic modification	Genetic background	Age of AD pathology first appear (month)	AD related pathology
1	Tg2576 (APPSwe)	APP KM 670/671NL	B6; SJL	5 m	Extensive amyloid pathology
2	JNPL3(P301L)/Tg(Prnp-MAPT*P301L)	htau with P301L mutation	B6, DBA/2, SW	4.5 m/homo. and 6.5 m/hemi.	Homozygous develops human-like tauopathies faster than the hemizygous model
3	APP/PS1	Chimeric Mo/HuAPP695swe and a mutant hPS1 (PS1-dE9)	B6;C3	6 m	Early-onset of amyloid plaque
4	ARTE10 (APP-PS1)16347	hAPP 695-a.a. isoform with Swedish mutation and hPS1 with M146V mutation	B6	3 m/homo. and 5 m/hemi.	Robust and reliable plaque pathology
5	APPSWE-TAU-2469	hAPP695-a.a. isoform and hMAPT P301L mutation	B6,DBA/2, SJL, SW	3 m	similar plaque pathology to Tg2576 with more extensive neurofibrillary tangles than the JNPL3.
6	3xTg-AD	APP KM 670/671NL, MAPT P301L, and PS1 M146V	B6;129	6 m	age-related and progressive plaques and tangles. tau pathology at 12 m. Synaptic dysfunction occur before plaques and tangles
7	5xFAD, TG6799	hAPP695 isoform with Swedish KM 670/671NL, Florida (I716V), London (V717I), hPS1 with M146L and L286V mutations	B6, B6SJLF1	2 m	Amyloid pathology; reduced synaptic marker protein levels
8	APOE3	hAPOE3 from the endogenous APOE locus	B6	NA	NA
9	APOE4	hAPOE4 allele from the endogenous APOE locus	B6	4 m	Decreased levels of total cholesterol, LDL and HDL
10	Trem2*R47H	an R47H point mutation and two silent mutations (lysine AAG > AAA and alanine GCC > GCA) into mTrem2	B6	NA	NA
11	Trem2*Y38C	Y38C point mutation	B6	NA	TREM2-deficient microglia fail to proliferate and cluster around plaques
12	APOE4/Trem2*R47H	hAPOE4 knock-in mutation and R47H point mutation	B6	NA	NA
13	HApp/APOE4/Trem2 *R47H	triple mutants with hAPOE4, R47H mutation mTrem2, and a hAPP within the mApp	B6	NA	the human Aβ generated by these mice is more aggregation-prone than the endogenous mouse Aβ

#### Mice With the Familial APP Mutations V717F (Indiana) and K670N/671L (APPswe, Swedish)

The first Aβ plaque-developing mouse model is APP (V717F), which progressively develops extracellular thioflavin S-positive Aβ deposits, neuritic plaques, synaptic loss, astrocytosis, and microgliosis (Games et al., 1995). This model can be used for testing therapeutic drugs targeting amyloidosis. The commonly studied Swedish APP mutation (APPswe) K670N/M671L carries a transgene coding for the 695-amino acid isoform of human Aβ precursor protein bearing the Swedish mutation (Sturchler-Pierrat et al., 1997). This mouse model expresses high concentrations of the mutant Aβ, develops significant amyloid plaques, and displays memory deficits. It is useful for studying APP expression, amyloid plaque formation, neuronal decline, and memory loss associated with AD, as well as drug discoveries. Recently, Xu et al. (2015) achieved the amyloid deposition using murine genes carrying the APPswe mutation, indicating murine Aβ peptides can produce amyloid deposits that morphologically resemble those found in human AD.

#### Mice With the Familial FTLD MAPT Mutation P301L or Human Tau

The first NFT-developing mouse was achieved by expressing the familial FTLD MAPT mutation P301L under the control of the mouse prion promoter (Lewis et al., 2000). Allen et al. (2002) used mouse Thy1.2 promoter to reach a 2-fold increase in the expression of P301S mutant FTLD-tau compared with endogenous tau, with NFTs forming at 5 months of age. The first human MAPT transgenic model (ALZ7) with the human THY1.2 promoter expressed only a low level of the transgenic gene but achieved deposition of hyperphosphorylated tau in the somatodendritic domain (Gotz et al., 1995). The rTg4510 model uses a reversible binary transactivator system to achieve a high level of tau expression (13-fold) with P301L, NFT-like lesions, neuronal loss, cognitive impairment, and brain atrophy at an earlier time frame (Santacruz et al., 2005). This mouse develops progressive intracellular tau aggregations in the corticolimbic areas and forebrain atrophy. The human Tau (hTau) mice (Andorfer et al., 2003) were generated by crossing 8c mice expressing human 3R and 4R tau isoforms (Duff et al., 2000) with tau knockout (KO) mice generated by targeted disruption of exon one on the MAPT gene (Tucker et al., 2001). This mouse model expresses all six isoforms of hTau but lacks mouse tau. It develops age-associated tau pathology that appears most severe in the neocortex and hippocampus. No tau pathology was found in both 8c mice and tau KO mice. Recently, Saito et al. (2019) used a homologous recombination approach to replace the entire murine *Mapt* gene with the human ortholog to create a *MAPT* knock-in (KI) mouse model that expresses all six tau isoforms present in humans. They cross-bred the *MAPT* KI mice with single *App* KI mice to generate the APP/MAPT double knock-in (dKI) mice that exhibit higher tau phosphorylation than the single MAPT KI mice (Saito et al., 2019).

#### APOE-Target Replacement Mice (APOE-TR Mice)

The APOE family consists of three isoforms: APOE2, APOE3, and APOE4, with APOE4 being the greatest genetic risk factor for AD (Kim et al., 2009). APOE-target replacement mice (APOE-TR Mice), in which the m-APOE coding sequence is replaced by that of an h-APOE allele, display alterations in synaptic number and structure, network connectivity, and behavior based on the specific APOE isoform expressed (Ji et al., 2003; Wang et al., 2005; Tai et al., 2011; Zhu et al., 2012; Dumanis et al., 2013; Koutseff et al., 2014; Neustadtl et al., 2017; Lewandowski et al., 2020). These mice exhibit isoform-specific differences in lipid physiology and synaptic function. Mice with h-APOE4 exhibit earlier and more severe AD pathology and memory decline (Bour et al., 2008; Sun et al., 2017; Lewandowski et al., 2020).

#### Triggering Receptor Expressed on Myeloid Cells 2 Gene-Modified Mice

Triggering receptor expressed on myeloid cells 2 (TREM2) is expressed in microglia, and its genetic variants R47H and Y38C are linked to AD, frontotemporal dementia, and Nasu-Hakola disease, which is an early onset of dementia characterized by white matter pathology (Yaghmoor et al., 2014). While Trem2 variant R47H is largely associated with late-onset AD, Trem2 variant Y38C is associated with the development of early onset dementia (Jadhav et al., 2020). Both Trem2R47H and Trem2Y38C mice were generated using the CRISPR/Cas9 technique to introduce the point mutations of Trem2R47H and Trem2Y38C. TREM2 R47H homozygous mice exhibit a novel splice variant resulting in partial expression of mRNA and protein in the brain (Xiang et al., 2018). While mice harboring the Trem2 Y38C exhibited normal expression levels of TREM2, alterations were observed in the expression of neuronal and oligodendrocyte/myelin genes, along with regional decreases in synaptic protein levels, particularly in the hippocampus (Jadhav et al., 2020).

#### APP/PS1 Mice

In addition to the “single-gene models” described above, combinations of AD-related genes have also been introduced in mice using transgenic technology. These combinatorial genetic models present greater phenotypical diversity, and thus offer more options for preclinical studies. The APP/PS1 mouse model was generated by administration of both APPswe mutant (K595N/M596L) and the ΔE9 mutant of presenilin 1 (PS1), which is an essential component of γ-secretase, the enzyme responsible for APP cleavage. Mutations in PS1 lead to dominant inheritance of early-onset FAD (Jankowsky et al., 2001). The mice develop Aβ deposits in the brain by 6–7 months of age, with 15-month-old females presenting a 5-fold (Aβ42) and 10-fold (Aβ40) increase in Aβ deposits in the cerebellum compared to males (Jankowsky et al., 2004; Ordonez-Gutierrez et al., 2016).

#### The 3 × Tg Strain

Although there are no reports that APP, PS1, and tau mutations occurring simultaneously in humans, the 3 × Tg strain is the most widely used model that presents aggregated Aβ and synaptic dysfunction. This model is created by co-injecting two constructs expressing APPswe and P301L mutant tau into oocytes obtained from PS1 M146V KI mice. These triple transgenic mice express mutant APP, PSEN2, and MAPT and show age-dependent accumulation of Aβ plaques and neurofibrillary tangle-like pathology, starting around 4 months of age (Grueninger et al., 2010).

#### The 5 × FAD Strain

The 5 × FAD strain combines the APPswe mutation with the Florida (I716V) and London (V717I) mutations of APP, as well as the M146L and L286V mutations of PSEN1. These mice show progressive cognitive deficits with several pathological hallmarks of AD, such as Aβ plaques, gliosis, synaptic degeneration, and neuronal loss, and develop tau pathology (Oakley et al., 2006).

#### The APOE4/TREM2R47H Mouse

This double mutant strain carries a humanized APOE4 knock-in mutation and a CRISPR/cas9-generated R47H point mutation of the Trem2 gene. This strain does not produce any severe phenotypes, even late in life, allowing a better understanding of the effect of AD risk factors in the context of aging (Kotredes et al., 2021).

#### Knockout Mice

Several AD-related KO mice are generated for understanding the pathophysiological role of AD-related proteins, including APP, MAPT, BACE1, APOE, PSEN1, PSEN2, and Trem E (Table 2).

**TABLE 2:** List of AD-related gene knockout mice models.

#	Strain name	Genetic modification	Genetic background	Phenotype
1	APP−/−	APP gene was disrupted by inserting a stop codon into the first exon through homologous recombination	B6	Deficits in forelimb grip strength and locomotor activity and an age-related deficit in retention of memory for an aversive experience (Senechal et al., 2008)
2	PS1	Psen1 knock-out	B6	A drastic reduction in neural progenitor cells in the embryo with death occurring minutes after being born
3	MAPT−/−	A PGK-neo cassette is inserted into the first exon of tau, only short fragments incapable of binding to MTs	129 × B6	Not much evidence of brain dysfunction (Dawson et al., 2001)
4	PSEN1−/−	Disruption of exons 1 to 3	129 × B6	Perinatal lethal in homozygous animals which die shortly after birth (Shen et al., 1997)
5	PSEN2−/−	The replacement of exon 5 by the hygromycin cassette results in a frame shift between exons 4 and 6.	129 × B6	Do not display any gross brain abnormalities, astrogliosis, or behavioral abnormalities by 12 m. APP processing is not affected (Herreman et al., 1999)
6	APOE2-1547	Homozygous for a human APOE2 gene targeted replacement of the endogenous mouse APOE gene	129 × B6	Hyperlipoproteinemia with elevated plasma cholesterol and triglyceride levels, decreased clearance of vLDL particles, and spontaneous atherosclerotic plaques on a normal diet, exacerbated by a high fat diet
7	APOE3 1548	Homozygous for a human APOE3 gene targeted replacement of the endogenous mouse APOE gene	129 × B6	Increased risk of atherosclerosis and hypercholesterolemia compared with wild type mice on a high fat diet, but not on a normal diet
8	APOE4-1549	Homozygous for a human APOE4 gene targeted replacement of the endogenous mouse APOE gene	129 × B6	At increased risk of atherosclerosis compared with wild-type animals or mice expressing human APOE3
9	Trem2 KO (KOMP)	The entire coding region of the Trem2 gene was replaced by Velocigene cassette ZEN-Ub1 (lacZ −p(A)−loxP-hUbCpro-neor-p(A)-loxP)	B6	Trem2−/− microglia show less proliferative activity and less pronounced changes in morphology than do wild-type microglia after an excitotoxic insult no behavioral and cognitive deficit (Kang et al., 2018)
10	BACE1−/−	Targeted deletion in the mouse gene β-site APP cleaving enzyme 1	129 × B6	Do not display any gross physical or behavioral abnormalities (Cai et al., 2001)

APP KO mice display deficits in forelimb grip strength and locomotor activity and an age-related deficit in retention of memory for an aversive experience (Senechal et al., 2008). MAPT KO mice have been reported to show less evidence of brain dysfunction (Harada et al., 1994; Dawson et al., 2001; Morris et al., 2011). PSEN1 KO mice exhibit perinatal lethality in homozygous animals, which die shortly after birth (Shen et al., 1997). PSEN2 KO mice are viable and normal in growth and size and do not display any gross brain abnormalities, astrogliosis, or behavioral abnormalities by 12 months of age, and no deficit in APP processing (Herreman et al., 1999). APOE KO mice display poor lipoprotein clearance with subsequent accumulation of cholesterol-ester-enriched particles in the blood (Piedrahita et al., 1992). The systemic proinflammatory status of APOE KO mice also makes them good candidates for studying risk factors for AD (Lo Sasso et al., 2016). TREM KO mice show no behavioral and cognitive deficit (Kang et al., 2018). BACE1 KO mice do not display any gross physical or behavioral abnormalities (Cai et al., 2001).

#### Chemical-Induced AD Models

Alzheimer’s disease models can also be generated by chemical induction. Synthetic Aβ and tau aggregates have been intraperitoneally injected to induce cerebral amyloids and intracerebral tauopathy (Gotz et al., 2001; Clavaguera et al., 2014). Intracranial injection of okadaic acid, a protein phosphatase inhibitor, increased tau phosphorylation and protein aggregation in distinct brain regions (Baker and Gotz, 2016). Intracranial injection of synthetic Aβ aggregates into P301L tau transgenic mice can accelerate NFT formation (Peeraer et al., 2015). Brain lysates from both transgenic mice and patients with AD also induce nucleation of protein aggregation along with neuronal projections in healthy mice or mice with preexisting AD pathology (Bolmont et al., 2007; Clavaguera et al., 2009; He et al., 2018). Lipopolysaccharide (LPS) acts as a Toll-like receptor 4 ligand to activate microglia to produce proinflammatory cytokines such as TNF-α, IL-1β, prostaglandin E_2_ (PGE_2_), and nitric oxide (NO) in the central nervous system (Heneka et al., 2015). The administration of LPS to animals induces cognitive impairment (Shaw et al., 2001; Choi et al., 2012) and high levels of Aβ_1–42_ (Zhao et al., 2019).

Other chemicals used for the induction of cognitive impairment include heavy metals (e.g., aluminum, cobalt, and cooper), scopolamine, ethanol, colchicine, an excitotoxin, streptozotocin, and sodium azide, among others, and have been nicely summarized in the review (More et al., 2016; Götz et al., 2018).

#### Injury-Based and Trauma-Based Models

Brain injury is associated with elevated Aβ levels and tau phosphorylation (Yu et al., 2012), but not the formation of plaques and NFTs. In transgenic hTau and 3xTg mice, brain injury accentuates the development of tau pathology and Aβ accumulation (Tran et al., 2011; Ojo et al., 2013).

#### Next-Generation AD Models

There is no AD mouse model that recapitulates all aspects of human AD. Even with the high levels of amyloid protein, the mice still do not display human-like cognitive deficits. The Aβ plaques in mice are often diffuse or exhibit fewer crosslinking fibrils even when they appear condensed. The tau pathology also shows a certain difference from humans, with a wide and uncontrollable range of expression levels in some AD model mice. Because there are dozens of different genes that are associated with AD, the different combinations of mutations in these genes, in conjunction with varying environmental stimulators, will contribute to each unique AD case (Naj and Schellenberg, 2017). Furthermore, the offspring of AD transgenic and wild mice are more likely to develop memory loss, indicating there are AD-associated genetics or environmental factors yet to be elucidated, which drives the continuing efforts for better mouse models. In 2016, the NIH started the MODEL-AD consortium to engineer mice with different genetic mutations associated with early- or late-onset AD (model-ad.org).

### Rat Models

Compared to mice, rats are easier to handle and have larger brain sizes for easier surgical operation and imaging analysis (Ellenbroek and Youn, 2016). Both genetic and non-genetic rat AD models have been developed (Table 3). However, unlike transgenic AD mouse models, not as many AD rat models are available for scientific research and rats appear to be more resilient to AD pathology than mice (Charreau et al., 1996).

**TABLE 3:** List of major AD rat models.

#	Strain name	Genetic modification	Genetic background	Age of AD pathology first appear (month)	AD related pathology
1	TgAPPswe	hAPP KM 670/671NL	Fischer-344	NA	No extracellular Aβ deposits
2	Tg6590	hAPP KM 670/671NL	Sprague-Dawley	11 m	The levels of both Aβ species are increased 65% in hippocampus and 40% in cortex of 11-month-old animals.
3	McGill-R-Thy1-APP	hAPP751 with K670N/671L and V717F	HsdBrl:WH Wistar	6–9 m	Homozygotes show age-dependent accumulation of Aβ plaques, gliosis, cholinergic synapse loss. Intracellular Aβ inclusions appears at postnatal day 7 and Aβ plaques at around 6–9 m
4	Tg1116	hAPP minigene containing K670N/671L and V717F	Sprague-Dawley	NA	NA
5	APP21	hAPP double mutant construct containing K670N/671L and V717F	Fischer-344	NA	NA
6	APP31	hAPP double mutant construct containing the K670N/671L and V717F	Fischer-344	NA	NA
7	Tg478/Tg1116	hAPP with K670N/671L and V717F	Sprague-Dawley	17-18 m	Aβ for amyloid deposition
8	TgF344-AD	APPswe and PS1ΔE9	Fischer-344	6 m	Age-dependent accumulation of Aβ plaques in hippocampus and cortex with age-dependent cerebral amyloidosis that precedes tauopathy, gliosis, apoptotic loss of neurons in the cerebral cortex and hippocampus and cognitive dysfunction. Tau pathology is reported
9	UKUR25	hAPP with K670N/671L and V717F and PS1 (M146L)	Wistar	NA	Intracellular accumulation of Aβ in hippocampus and cortex without extracellular amyloid
10	PS/APP (Tg478/Tg1116/Tg11587)	hAPP695 with K670N/671L and V717F and PSEN1 with ΔE9 (4.6 kb deletion of exon 9)	Sprague-Dawley	7 M	Aβ deposition
11	SHR24	Human non-mutated truncated tau encompassing 3R domains and a proline-rich region (3R tau151-391)	SHR	9 m	Age-dependent progressive neurofibrillary degeneration in the isocortex

#### Rat Models With the Familial Swedish and Indiana Mutations

The APP transgenic rats appear to have lower expression levels of the APP transgene than the mouse AD model (Benedikz et al., 2009). TgAPPswe is the first APP transgenic rat that overexpresses human APP with Swedish mutation (K670N and M671L) (Ruiz-Opazo et al., 2004), with only a 56.8% increase in the expression level of APP mRNA, 21% increase for Aβ42, and 6% for Aβ40 in the brain. No AD-related pathology was found in these animals up to the age of 18 months. The Tg6590 rat generated by Fokesson et al. is another model that carries human APP with the Swedish mutation. The levels of both Aβ species are increased by 65% in the hippocampus and 40% in the cortex of 11-month-old animals. The rats display learning and memory deficits in the Morris water maze at 9 months and altered spontaneous behavior measured in open field (Kloskowska et al., 2010). The McGill-R-Thy1-APP rat model expressed hAPP751 bearing the Swedish and Indiana mutations, with intracellular Aβ inclusions detected as early as postnatal day 7 and Aβ plaques at 6–9 months of age (Leon et al., 2010). The Tg1116 rats express a human APP minigene containing both the Swedish and Indiana familial AD mutations (Flood et al., 2009). APP21 and APP31 express a human APP double mutant construct containing the Swedish and Indiana AD mutations driven by the ubiquitin-C promoter. The APP transgene is reported to be expressed in the brain, in neuronal but not glial cells (Agca et al., 2008). No pathological or behavioral studies have been published yet. The double homozygous Tg478/Tg1116 rats were generated by crossing Tg478 which expresses human APP with the Swedish mutation (Flood et al., 2009) and Tg1116. The rats produce sufficient levels of Aβ for amyloid deposition to occur by the age of 17–18 months (Flood et al., 2009).

#### APPswe and PS1ΔE9/TgF344-AD Rats

TgF344-AD rats co-express APPswe and PS1ΔE9 transgenes and present with age-dependent cerebral amyloidosis that precedes tauopathy, gliosis, apoptotic loss of neurons in the cerebral cortex and hippocampus, and cognitive dysfunction (Cohen et al., 2013).

#### Rat Model With the Familial APP Mutations V717F (Indiana) and Swedish APP (APPswe) K670N/671L and Human PSEN1 With the Finnish M146L Mutation

UKUR25 rats express human APP containing the Swedish and Indiana (V717F) mutations, and mutated PS1 (M146L). The main pathological feature was an intracellular accumulation of Aβ in neurons of the hippocampus and cortex without extracellular amyloid up to 24 months of age. Mild impairment in acquisition learning was found in 16-month-old male rats, with an increase in tau phosphorylation at S396 and S404 ERK2 sites (Echeverria et al., 2004a,b).

#### PSAPP (Tg478/Tg1116/Tg11587) Rats

The PSAPP model rats express hAPP695 carrying the Swedish and London (K670N/M671L and V717I, respectively) mutations together with PSEN1 carrying the Finnish mutation (PS1, ΔE9) and develop Aβ deposition around 7 months of age (Flood et al., 2009). This strain was created by crossing double homozygous Tg478/Tg1116 rats with Tg11587 that carries a human PS-1 transgene with the familial AD mutation M146V. The homozygous rats produce sufficient levels of Aβ for amyloid deposition to occur by the age of 7 months. The triple homozygous transgenic rat, Tg478/Tg1116/Tg11587, has also been called the PSAPP rat. The compact amyloid deposits were found to be associated with activated microglia, reactive astrocytes, and phosphorylated tau immunoreactivity.

#### AD-Tau Rat Model

Overexpression of human non-mutated truncated tau encompassing 3R domains led to the first rat model of progressive cortical neurofibrillary degeneration (Filipcik et al., 2012). This transgenic rat expresses a truncated form of the human tau protein (truncated at amino acid positions 151–391), which is found in the brains of sporadic AD patients (Benedikz et al., 2009).

#### Chemical-Induced AD Rat Models

The aforementioned chemicals used to generate AD mouse models can also be employed in rats to create AD phenotypes (More et al., 2016; Götz et al., 2018).

### Large Animal AD Models

Non-human primates such as rhesus macaques and marmosets are not known to develop AD but do accumulate Aβ deposits and show tauopathy in their aged brains (Paspalas et al., 2018; Haque and Levey, 2019; Arnsten et al., 2021b; Datta et al., 2021; Leslie et al., 2021). Intracranial injection of Aβ_42_ and thiorphan, an inhibitor of neprilysin that is responsible for Aβ clearance, has been employed to generate an AD model in middle-aged (16–17 years) rhesus monkeys (Li et al., 2010). Significant intracellular accumulation of Aβ was found in the neurons of the basal ganglia, cortex, and hippocampus, accompanied by neuronal atrophy and loss. Two injections of an adeno-associated virus expressing a double tau mutation (AAV-P301L/S320F) in the left hemisphere of rhesus monkeys result in misfolded tau propagation similar to that in humans. Tau spreading is accompanied by robust neuroinflammatory response driven by TREM2 + microglia, with biomarkers of inflammation and neuronal loss in cerebrospinal fluid and plasma (Beckman et al., 2021).

Other non-primate large animals used for AD modeling include domestic animals such as dogs and cats as well as farm animals including pigs, sheep, and cows. Aged dogs develop plaque pathology and cerebral amyloid angiopathy (Yu et al., 2011), as well as a dementia-like syndrome resembling human AD (Prpar Mihevc and Majdic, 2019; Abey et al., 2021). Tau dysfunction and tangles have been reported and associated with cognitive decline (Yu et al., 2011; Schmidt et al., 2015; Smolek et al., 2016). Cats also develop plaques, tangles, and brain atrophy along with cognitive decline as they age (Chambers et al., 2015; Fiock et al., 2020). Two transgenic pig models of AD have been reported using minipigs. The first one carries an hAPP transgene with the Swedish mutation driven by the human BDGFβ promoter, resulting in high levels of brain-specific Aβ expression (Kragh et al., 2009), and the second minipig model carries three copies of a transgene expressing the 695 variant of hAPP with the Swedish mutation and a human PSEN1 transgene with the M146L mutation (Jakobsen et al., 2016). Intraneuronal accumulation of Aβ_1–42_ was detected in two pigs: one at 10 months and one at 18 months. Plaque- and tangle-like pathologies have also been seen after traumatic brain injury (TBI) in pigs (Hoffe and Holahan, 2019). Tau pathology and Aβ plaques have been identified in aged sheep and goats as well (Braak et al., 1994).

## Preclinical PET Imaging in AD Animal Models

### β-Amyloid Imaging

The development and validation of the first-in-class Aβ PET radiotracer, the thioflavin T-derived Pittsburgh compound B ([^11^C]PIB or PIB), was a milestone in AD imaging. It not only allows the direct *in vivo* visualization and quantification of Aβ plaque in living subjects (Klunk et al., 2004) but also paves the road for the development and FDA approval of its ^18^F-labeled analog ([^18^F]flutemetamol), the stilbene derivative [^18^F]florbetaben, and the styrylpyridine derivative [^18^F]florbetapir, the use of which have become impactful in AD clinical trials and diagnosis. The intrinsic fluorescent characteristics of these imaging probes and their analogs allow for the microscopic assessment of their binding selectivity and binding preference to different forms of Aβ plaques and Aβ plaques at different locations (e.g., parenchymal and cerebral amyloid angiopathy, CAA) (Bacskai et al., 2003; Fodero-Tavoletti et al., 2012).

**FIGURE 2:**
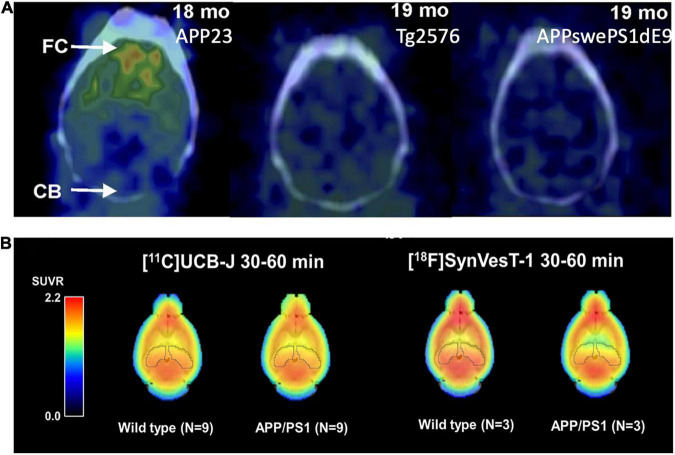
**(A)** [^11^C]PIB binding to Aβ deposits varies by mouse strains. APP23: Extensive Aβ deposits; Tg2576: Mild Aβ deposits; APP-swePS1dE9: Extensive Aβ deposits. This figure was adapted and modified from Snellman et al. J Nucl Med. 2013;54:1434-1441. **(B)** Uptake of the SV2A PET tracers [^11^C]UCB-J and [^18^F]SynVesT-1 in the brain of APP/PS1 and wild-type mice. The uptake of both tracers was lower in the hippocampus of APP/PS1 mice compared to wild-type controls.

Many Aβ imaging tracers have been evaluated using multiple different AD animal models, mainly in AD mice. PIB has been tested in AD mice of APPswe, APP/PS1, 3 × Tg, 5 × FAD, Tg2576, and APP23 (Ni, 2021). Initial reports on PIB binding in Tg2576 and APP/PS1 mice at advanced ages were negative, even with abundant Aβ pathology (Klunk et al., 2005; Toyama et al., 2005); while PIB binding in APP23 mice was positive (Maeda S. et al., 2007). These data led to the hypothesis that the paucity of high-affinity binding sites for PIB in murine Aβ plaques requires very high molar activity PIB for successful imaging in murine AD models. Snellman et al. (2013) compared PIB uptake longitudinally in the brains of multiple AD mouse models and found higher PIB uptake in the cortex of APP23 mice compared with the wild-type controls and no difference in APP/PS1 and Tg2576 mice with their corresponding controls, consistent with previous results from other groups (Figure 2A). They also compared the thioflavin-T staining patterns and found that APP23 mice form large and compact human-like Aβ deposits, whereas Tg2576 mice and APP/PS1 mice form sparse fibrillar deposits. The results suggest that PIB binding is highly dependent on the AD model and the associated higher-order fibrillar structure rather than simple β sheets. At a microscopic level, the Aβ plaques formed in early onset autosomal dominant AD and sporadic AD brains have different levels of non-fibrillar Aβ species (Querol-Vilaseca et al., 2019), and the Aβ deposits in familial AD, sporadic AD, and cerebral amyloid angiopathy manifest different conformations (Condello et al., 2018). Further understanding of the interactions of the imaging probes with amyloid plaques of different forms will help with the development of probes targeting the various forms of misfolded Aβ proteins in the brain (Biancalana and Koide, 2010).

One of the biggest advantages of small animal PET imaging is the longitudinal tracking of the pathogenesis and therapeutic effects of experimental drugs. This was demonstrated by the longitudinal PET imaging studies in AD animal models (Maeda J. et al., 2007; Deleye et al., 2017; Snellman et al., 2017). The challenges in imaging Aβ plaques in AD animal models are due to the different forms of plaques and disposition patterns in different animal models and at different ages of the same animals (Snellman et al., 2013). Other challenges are the quantification of the PET signals. For the quantitative analysis of human Aβ PET imaging data, the cerebellum was chosen as the reference region to generate distribution volume ratio (DVR) or standardized uptake value ratio (SUVR) because of the lack of specific binding of PIB in the human cerebellum (Lopresti et al., 2005; Price et al., 2005). However, there are emerging effective drugs targeting other pathological pathways and that do not alter Aβ plaque levels, e.g., Fyn inhibitor and mGluR5 silent allosteric modulator (SAM) (Kaufman et al., 2015; Haas et al., 2017). Thus, the objective assessment of their treatment effects needs different imaging biomarkers that are closely related to synaptic/functional recovery rather than Aβ plaque levels. The current consensus considers Aβ oligomers as the primary cause of the neurotoxicity derived from abnormal amyloidosis. Thus, the development of an imaging agent targeting Aβ oligomers is highly desirable, albeit challenging.

Large molecules such as antibodies have been developed for Aβ PET imaging. Sehlin et al. (2016) and Fang et al. (2019) engineered the ^124^I-labeled Aβ antibodies [^124^I]mAb158 and Di-scFv [^124^I]3D6-8D3, respectively, to detect the soluble Aβ in the tg-ArcSwe (AβPP E693G) and Swedish (AβPP KM670/671NL) mouse models with clearly visualized Aβ in the brain. The brain PET imaging shows a correlation between the PET signal and the levels of soluble Aβ aggregates. An increased SUVR of 2.2–3.5 in AD mice brains was reported compared to the wild-type brains. The evaluations of other amyloid imaging tracers in AD models have been summarized nicely in a recent review (Ni, 2021).

### Tau PET Imaging

Targeting another hallmark of AD, tau neurofibrillary tangle is an extremely exciting area of PET tracer development. Tau is an axonally enriched microtubule-associated protein (MAP) that accumulates in the temporal and parietal neocortex in AD brains (Hung et al., 2016; DeTure and Dickson, 2019). Tau exists as six different isoforms, which contain either 3 or 4 microtubule-binding repeats (3R or 4R). The hyperphosphorylation and aggregation of tau with different repeats are involved in different neurodegenerative diseases, e.g., AD (3R/4R), Pick disease (3R), and progressive supranuclear palsy (4R). Postmortem histopathological studies demonstrated that NFTs are a better index of disease severity and progression than Aβ for patients with AD (Shoghi-Jadid et al., 2002). Tau pathology appears earlier than the Aβ plaque in human brains (Arnsten et al., 2021a). Tau-PET imaging allows the detection of tauopathy and highly predicts subsequent cognitive decline in both asymptomatic and symptomatic individuals (Leuzy et al., 2019; Wang and Edison, 2019; Beyer and Brendel, 2021).

#### First-Generation Tau Radioligands

[^18^F]FDDNP is the first PET tracer to visualize both amyloid plaques and tau tangles in living humans (Shin et al., 2011). Kuntner et al. (2009) compared 13–15-month-old age-matched wild-type litter mates with Tg2576 mice and found no difference in regional brain kinetics and DVR values. Later, the so-called first generation tau radioligand including [^18^F]THK523, the first tau selective tracer (Okamura et al., 2005), and other THK family tracers ([^18^F]THK5105, [^18^F]THK5117, [^18^F]THK5317, and [^18^F]THK5351) were developed and evaluated in human and AD mouse models. Fodero-Tavoletti et al. (2011) found higher retention of [^18^F]THK523 in the brains of rTg4510 mice compared with their wild-type littermates or 12-month-old APP/PS1 mice. Brendel et al. (2016) investigated [^18^F]THK5117 in Tau-P301S mice (PS19) and bigenic GSK-3β × Tau-P301L (biGT) mice and found increased SUVR in the brain stem of aged P301S mice and the entorhinal/amygdaloidal areas of biGT mice. In a separate study, the same group conducted a head-to-head comparison of [^18^F]T807 and [^18^F]THK5117 in Tau-P301S (P301S) mice (Brendel et al., 2018). Significantly elevated [^18^F]T807 than [^18^F]THK5117 uptake in the brainstem of P301S mice was evident at 6 months, and this increased further at 9 months. Thus, [^18^F]T807 appeared to be more sensitive than [^18^F]THK5117 to detect tau pathology in this model. Recently, [^18^F]THK5351 PET signal was found to correlate well with histological and biochemical tau changes, as well as motor, memory, and learning impairment, in P301S tau mice from 8 months over time (Moreno-Gonzalez et al., 2021).

Nevertheless, due to the off-target binding to monoamine oxidase-B (MAO-B), the THK family tracers are deemed to have limited utility in imaging tauopathies in AD (Ng et al., 2017; Murugan et al., 2019; Bao et al., 2021).

[^11^C]PBB3 is a pyrinated phenyl- and pyridinyl-butadienyl-benzothiazole and has been clinically used for *in vivo* detection of tauopathies in the human brain. [^11^C]PBB3 has been tested in the rTG4510 mouse (Ishikawa et al., 2018) and the PS19 transgenic mouse model (expressing 4R tau pathology) (Maruyama et al., 2013). Ni et al. (2018) compared [^11^C]PBB3 in PS19 and rTg4510 models and found increased binding *in vivo* in the neocortex and hippocampus of rTg4510 mice. In contrast, *in vitro* [^11^C]PBB3 binding was elevated in the brain stem but not in the hippocampus of PS19 mice. [^18^F]PM-PBB3, an ^18^F-labeled derivative of [^11^C]PBB3, has been demonstrated to detect significant tau deposits as measured by SUVR in the rTg4510 mice as early as 6 months of age (Weng et al., 2020). Recently, McMurray et al. reported the synthesis of [^11^C]LM229 based on the backbone of PBB3. [^11^C]LM229 showed high specificity for 4R tau aggregated in the brain sections of P301S tau mice and truncated human 151–351 3R (SHR24) and 4R (SHR72) tau aggregates in tau transgenic rat brain sections. Preliminary PET studies with [^11^C]LM229 in both WT and transgenic P310S tau mice confirmed BBB penetration by the radiotracer with maximum brain uptake (%ID/g max; WT = 1.56, P301S = 2.38) within the first minute, followed by washout during the 90-min scan (McMurray et al., 2021).

The most widely studied first-generation tau radioligand [^18^F]flortaucipir ([^18^F] T-807 and [^18^F]AV-1451) did not show any different retention in the cerebrum of the P301L tau transgenic mice compared to wild-type mice (Xia et al., 2013; Declercq et al., 2016), which was attributed to the use of transgenic mice expressing structurally different tau deposits in the animals than in humans (Duyckaerts et al., 2008).

#### Second-Generation Tau Radioligands

Second-generation radiotracers with improved signal-to-noise ratio, less off-target, and lower non-specific binding are now available for tau imaging research. These tracers include [^18^F]PI2620, [^18^F]MK6240, [^18^F]GTP1, [^18^F]RO-948 (RO6958948), [^18^F]JNJ311 (JNJ64349311), and [^18^F]JNJ-067 (JNJ-64326067). Preliminary studies have been carried out in humans and healthy mice with promising results regarding the binding selectivity, affinity, and stability (Bao et al., 2021). So far, these tracers have not been tested in AD animal models.

### PET Imaging of Glucose Metabolism

Brain [^18^F]FDG PET primarily indicates synaptic activity. [^18^F]FDG uptake strongly correlates at autopsy with levels of the synaptic vesicle protein synaptophysin (Rocher et al., 2003). The degree and regional extent of hypometabolism measured by [^18^F]FDG-PET roughly correlate with the overall severity of cognitive impairment in AD. There is a close correlation between the regional accumulation of a tau-PET tracer ([^18^F]AV1451) and [^18^F]FDG hypometabolism (Rubinski et al., 2020). Along with amyloid imaging, [^18^F]FDG PET has been applied in multiple AD rodent models such as APPswe (Tg2576), 5 × FAD, APP/PS1, 3 × Tg, Tg4-42, TASTPM mice, and McGill-R-Thy1-APP rats (Waldron et al., 2015b; Bouter et al., 2018; Bouter and Bouter, 2019). Varying [^18^F]FDG PET results were found in Tg2576 mice. No differences in cerebral glucose metabolism were found in Tg2576 compared to WT mice in Kunter’s study (Kuntner et al., 2009), while Luo et al. (2012) found an increase in the FDG uptake in 7-month-old Tg2576, and Coleman et al. (2017) reported reduced FDG uptake in 18-month-old mice. Two separate [^18^F]FDG PET studies using 12-month-old APPPS1-21 mice reached the same conclusion that FDG uptake was reduced in the brain (Waldron et al., 2015a; Takkinen et al., 2017). Using APP/PS1 mice, both Poisnel et al. (2012) and Li et al. (2016) showed an age-dependent increase in glucose metabolism. In addition, PS2APP mice showed increased [^18^F]FDG uptake at 5 and 16 month (Brendel et al., 2016) and TASTPM mice were found to have decreased FDG uptake at 9 and 14 months of age (Waldron et al., 2015a,^2017^; Deleye et al., 2016). Contradictory results were reported in 5xFAD mice, with Rojas et al. (2013) reporting increased uptake of [^18^F]FDG in 11-month-old 5xFAD, and Macdonald et al. (2014) showing decreased uptake in 13-month-old mice. Sancheti et al. (2013) also reported decreased FDG uptake in 3xTg mice.

Clinical FDG PET imaging studies have shown promise in detecting early AD as neurodegeneration in certain brain regions (temporoparietal predominantly) is reflected by hypometabolism of FDG (Cohen and Klunk, 2014), and the hypometabolism pattern could serve as a predictive biomarker for conversion from MCI to AD dementia (Sala et al., 2020) earlier than MRI (Laforce<suffix>Jr.</suffix>, Soucy et al., 2018). However, there has been no suitable method to distinguish the FDG signal contributed by neuronal activity and immune cell activation, and thus the FDG PET signal could theoretically be influenced by two opposing forces, i.e., hypometabolism and neuroinflammation, at certain stages of AD pathogenesis and progression. With the recent development of PET imaging methods for synapse density (see section “PET Imaging of Synaptic Vesicle Glycoprotein 2A” for SV2A PET) and neuroinflammation (see section “PET Imaging of Neuroinflammation” for PET imaging of neuroinflammation), we are at a stage where we could potentially quantitatively attribute the FDG signals to synaptic and glial activities. This is of relevance in cases of MCI patients who show a positive correlation between Aβ PET and FDG PET.

### PET Imaging of Synaptic Vesicle Glycoprotein 2A

Synaptic Vesicle Glycoprotein 2A is ubiquitously expressed in the neurons of the central nervous system and is widely used as one of the synaptic density biomarkers. Loss of synapses in the hippocampus and prefrontal cortex is implicated as an early pathological event in AD before the appearance of Aβ plaques and tau tangles and increasingly worsened during AD progression (Cai et al., 2019; Jackson et al., 2019).

[^11^C]Levetiracetam was first developed but was not pursued in further imaging study (Cai et al., 2014). Nevertheless, it encouraged the development of SV2A ligands with much higher affinities, including [^11^C]UCB-A (Estrada et al., 2016), [^18^F]UCB-H (Warnock et al., 2014; Bahri et al., 2017; Becker et al., 2017), and [^11^C]/[^18^F]UCB-J (Cai et al., 2019; Li et al., 2019b). Among these, [^11^C]UCB-J exhibited high brain uptake, fast and reversible tissue binding kinetics, and high specific binding signals in both non-human primates and humans (Finnema et al., 2016; Nabulsi et al., 2016). Most recently, [^18^F]SynVesT-1 (also known as [^18^F]SDM-8 (Li et al., 2019a) and [^18^F]MNI-1126 (Constantinescu et al., 2019) are developed and evaluated in non-human primates and humans (Li et al., 2021; Naganawa et al., 2021). Using APP/PS1 mice, Toyonaga et al. showed decreased [^11^C]UCB-J uptake as compared to the WT mice, and treatment with the tyrosine kinase Fyn inhibitor saracatinib reversed this effect (Toyonaga et al., 2019). Sadasivam et al. (2019, 2021) found a lower [^18^F]SynVesT-1 signal in the whole brain of APP/PS1 mice, compared with wild-type mice (Figure 2B). However, in a study using [^11^C]UCB-J in the tg-ArcSwe model and wild-type mice, a small but non-significant difference (∼5%) was found between the two groups, presumably due to large inter-animal variability (Xiong et al., 2021).

### PET Imaging of Neuroinflammation

Microglia are macrophages in the brain that play an important role in neuroinflammation in AD. PET imaging of biomarkers of microglia provides insights into the time course of AD pathology. However, the diverse phenotypes of activated microglia and their different roles over the course of the AD trajectory make it challenging to develop radiotracers specific for neuroinflammation in AD.

The 18kDa translocator protein (TSPO) has been widely studied as a biomarker for microglial activation for over 20 years. Early radiotracers had disadvantages of low brain penetrability, low binding affinity for TSPO, the short half-life of the radioisotope, and sensitivity of binding affinity to gene polymorphisms (Zhou R. et al., 2021). [^18^F]DPA714 is one of the more recent radiotracers developed for TSPO. [^18^F]DPA714 was evaluated in APP/PS1 mice at different months to determine the role of microglia in the pathogenesis of AD neuroinflammation (Hu et al., 2020). Higher [^18^F]DPA714 uptake was noted in the cortex and hippocampus of 12–13 and 15–16-months-old but not younger AD mice compared with control mice. Another longitudinal PET study in APP23 mice used [^18^F]GE180 for TSPO imaging and [^11^C]PIB for assessing amyloid deposition in *ex vivo* autoradiography experiments (Lopez-Picon et al., 2018). The APP23 model was chosen because of high [^11^C]PIB binding in the brain of model mice compared with other AD models such as APP/PS1. AD mice were imaged with [^18^F]GE-180 at 17, 20, and 26 months of age. The binding of [^18^F]GE-180 plateaued in the frontal cortex and hippocampus regions in the early stage of AD, but amyloidosis increased throughout the later stages of AD (17–26 months of age). Thus, [^18^F]GE-180 appeared to be useful for tracking TSPO/neuroinflammation in early-stage AD but not for monitoring disease progression.

Compared with TSPO, colony-stimulating factor 1 receptor (CSF1R) expression in the brain is predominantly localized to microglia and low in other cell types. [^11^C]CPPC was developed from a potent CSF1R inhibitor with an *IC*_50_ of 0.8 nM (Horti et al., 2019) and evaluated in a mouse model of AD-related amyloidosis-overexpressing APP with Swedish and Indiana mutations (Melnikova et al., 2013). [^11^C]CPPC had about 30% higher uptake in the cortex of AD mice compared with control mice at 40 min post injection. Significantly higher uptake in the hippocampus and cerebellum was also observed in the AD mice. Additionally, increased expression of CSF1R after LPS treatment and about 50% specific binding of [^11^C]CPPC in LPS-treated mice were observed relative to sham controls. Autoradiography studies with [^3^H]CPPC demonstrated the lack of specificity of [^3^H]CPPC in brain tissues of LPS-treated Sprague-Dawley rats (Knight et al., 2021). In another study, the imaging performance of [^11^C]CPPC was compared with that of [^11^C]GW2580 in mouse models of acute and chronic neuroinflammation and a rhesus monkey (Zhou X. et al., 2021). In WT vs. APP-KI mice, [^11^C]GW2580 demonstrated higher sensitivity than [^11^C]CPPC, shown by a greater increase in [^11^C]GW2580 uptake in the neocortex, forebrain, and striatum of APP-KI mice compared with that in WT based on SUVR measurements at 60–90 min. Blocking studies in the monkey showed higher specificity for [^11^C]GW2580 over [^11^C]CPPC.

TREM2 is a relatively new biomarker for microglial activation. Bispecific antibody scaffolds that bind to transferrin to enter the brain and to TREM2 were chemically conjugated and radiolabeled with relatively longer-lived radioisotopes. One example is ^124^I-mAb1729-scFv8D3_*CL*_, which was evaluated in Arc-Swe transgenic AD mice (Meier et al., 2021). While areas under the curve (AUC) for ^124^I-mAb1729-scFv8D3_*CL*_ at 24–72 h post injection were higher in caudate, cortex, thalamus, and hippocampus of AD mice compared with control mice, significant differences in SUVs were not observed for the individual imaging timepoints. However, *ex vivo* binding studies through autoradiography with the radiotracer showed significant differences between the animal models. The lack of a significant difference *in vivo* was then attributed to the increased blood residence time of ^124^I-mAb1729-scFv8D3_*CL*_. Thus, radiolabeling of smaller antibody fragments is desirable to address the slow pharmacokinetic issue.

Another new biomarker for AD is the purinergic P2X ligand-gated ion channel type 7 receptor (P2X7R), which is involved in triggering parts of the AD neurodegenerative processes. P2X7R activates microglia in acute AD models (Sanz et al., 2009) and increases the production of chemokines mediated by Aβ peptide in chronic AD models (Martin et al., 2019). [^18^F]JNJ-64413739 was evaluated in a rat model of acute neuroinflammation. The uptake of [^18^F]JNJ-64413739 was found to be elevated in the LPS-treated site of the rat brain compared with the contralateral hemisphere of the brain treated with PBS (Berdyyeva et al., 2019). Biomarkers for neuroinflammation, such as higher mRNA levels of P2X7R, TSPO, and Aif1, were associated with the LPS-treated site. It was acknowledged that LPS treatment as a model of neuroinflammation is considered extreme and that this novel tracer warrants evaluation in rodent models of AD and other neurodegenerative diseases. Other PET tracers for P2X7R and other biomarkers of neuroinflammation are reviewed by Zhou R. et al. (2021). So far, the development of neuroinflammation imaging agents has been focused on targeting microglial activation, largely ignoring the other glial cell types. It would be instrumental to be able to distinguish the protective microglial activation in early AD from the later destructive phenotype to guide the proper timing of anti-inflammatory treatments.

### PET Imaging of NMDA Receptors

Glutamate is the major excitatory neurotransmitter in the brain and acts on the ionotropic glutamate receptors (iGluRs) and metabotropic glutamate receptors (mGluRs) to regulate synaptic plasticity. iGluRs comprise three subfamilies: α-amino-3- hydroxy-5-methyl-4-isoxasolepropionic acid (AMPA) receptors, kainate receptors, and NMDARs (Traynelis et al., 2010). mGluRs are a family of G-protein-coupled receptors with 8 subtypes, mGluR1-8. Both iGluRs and mGluRs are found to be involved in synaptic malfunctions in AD (Avila et al., 2017; Foster et al., 2017; Wang and Reddy, 2017; Liu et al., 2019; Srivastava et al., 2020).

Several imaging tracers for glutamate receptors have been developed. So far, only the mGluR5 radiotracer [^18^F]FPEB has been evaluated in 5 × FAD mice (Lee et al., 2019), APP/PS1 mice (Varlow et al., 2020), and Tg-ArcSwe mice (Fang et al., 2017), with conflicting results: compared to wild-type animals, uptake of [^18^F]FPEB was found to be lower in 5 × FAD mice, with no difference in Tg-ArcSwe mice, and increased in APP/PS1 mice. Shimojo et al. (2020) observed that radioactivity signals derived from the other mGluR5 tracer (E)-[^11^C]ABP688 were unaltered relative to controls at 2 months of age in rTg4510 mice but then gradually declined with aging in parallel with progressive brain atrophy.

### PET Imaging of Cholinergic Targets

The deficit in cholinergic neurotransmission is a prominent pathophysiological feature in AD. Dramatic loss of cholinergic neurons located in the basal forebrain increased levels of α_7_ nicotinic acetylcholine receptor (α_7_ nAChR) (Ikonomovic et al., 2009; Marutle et al., 2013) and decreased levels of M1 muscarinic acetylcholine receptor (M1 mAChR) (Yi et al., 2020) were found in the cortical regions of human AD brains (Ferreira-Vieira et al., 2016). The following PET imaging agents for cholinergic targets have been developed: 1) [^11^C]NS14492 (Ettrup et al., 2011), [^11^C](R)-MeQAA (Nishiyama et al., 2015), and [^18^F]ASEM (Gao et al., 2013) for α7 nAChR; 2) [^11^C](+)3-MPB (Yamamoto et al., 2011) and [^18^F]fluorobenzyl-dexetimide (Rowe et al., 2021) for mAChR; 3) [^11^C]LSN3172176 for M1 mAChR (Nabulsi et al., 2019); and 4) [^11^C]MK-6884 M4 mAChR (Tong et al., 2020). Uptake of the α_7_ nAChR tracer [^11^C](R)-MeQAA was found to be increased in aged monkeys (Nishiyama et al., 2015), and lower uptake of the other α_7_ nAChR tracer [^18^F]ASEM was seen in aged TgF334 rats compared with wild-type rats (Chaney et al., 2021). No difference was noted in the brain uptake of the acetylcholine esterase tracer [^11^C]MP4A between the APP23 and wild-type mice at 10–13 months of age (Heneka et al., 2006).

### Other PET Radiotracers for AD

Altered expression of endogenous cannabinoid receptor 2 (CB2), histaminergic receptors, sigma receptors, adenosine receptors (A1A and A2A receptors), and enzymes (BACE1, caspase 3, aspartryl cathepsin, and TrkB/C), as well as abnormalities in dopamine and serotonin neurotransmission, have been noted in AD. These provide additional targets for PET radiotracer development for AD imaging. The CB2 radiotracers [^11^C]A-836339 have been tested in the LPS-induced neuroinflammation mouse model and the Appswe/PS1/dE9 mouse model (Horti et al., 2010), while [^18^F]JHU94620 has been tested in the LPS-induced neuroinflammation mouse model, which shows high-affinity binding to CB_2_R and sufficient selectivity over CB_1_R. A few tracers targeting caspase 3 and aspartryl capthepsin have been tested in AD model mice, with the majority of the tracers mainly tested in human subjects.

There has been great interest in imaging the compromised BBB in animal models of amyloidosis and patients with AD, as there is evidence of damaged BBB at the early stages of AD in patients and animal models. PET imaging of specific transporters and receptors expressed at the BBB was recently reviewed thoroughly by Ni^98^.

## Conclusion and Outlook

Alzheimer’s disease animal models have played essential roles in the development of PET radiotracers for imaging a diverse set of biological and pathological biomarkers in AD (Table 4). In turn, with well-validated PET tracers, PET imaging allows for longitudinal tracking of pathological phenotypes of AD in the same animals, boosting the statistical power in mechanistic studies of AD-related phenotypical and functional changes and facilitating the development of novel interventions through treatment effects monitoring. Currently, there is no perfect AD animal model that can fully recapitulate all features of human AD. However, with the rapid development in molecular biological technologies and our improved understanding of human AD etiology factors, we envision that the generation of more refined animal models with closer proximity to human AD pathogenesis will deepen our understanding of this devastating degenerative disease, further the development of biomarkers for preclinical diagnosis, and open new avenues for early and effective interventions.

**TABLE 4:** Summary of selected AD tracers.

Imaging target	Tracer structure	Imaging characteristics	Major finding	Reference
β-Amyloid	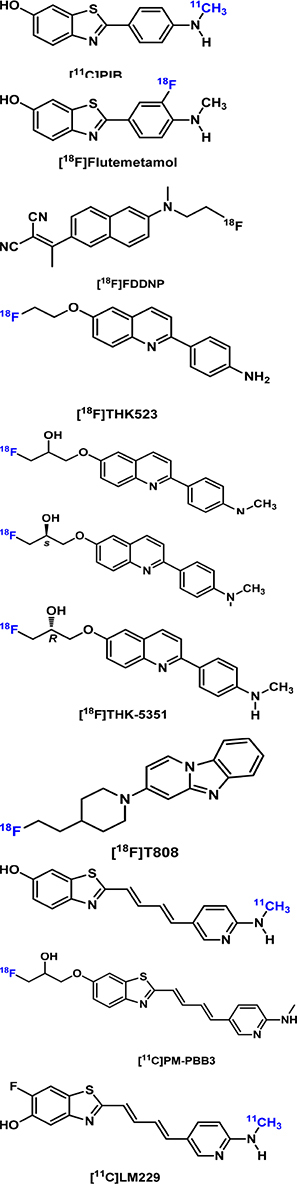	High binding affinity to large and compact Aβ deposits	Milestone in AD imaging	Rabinovici et al., 2007
		Similar to PIB but with higher non-specific binding in brain	FDA approved	Zeydan et al., 2021
TAU		Binds to both amyloid plaques and tau tangles	First PET tracer to visualize both amyloid plaques and tau tangles in living humans. No different uptake was found between Tg2576 and WT litter mates	Tauber et al., 2013
		Neurofibrillary tau tangles, off-target binding to MAO-B	First tau selective tracer. Higher retension in rTg4510 and APP/PS1 brains	Fodero-Tavoletti et al., 2011
			[18F]THK-5105 was tested clinically and evaluated in terms of whether it could selectively bind to tau aggregates in living patients with AD	Okamura et al., 2014
			Increased SUVR in subbrain regions in PS19 and biGT mice	Alzghool et al., 2021
			PET signal correlated well with histo and biochemical tau level in P301S tau mice	Rodriguez-Vieitez et al., 2017
			The most widely studied first-generation tau radioligand. Increased uptake in PS19 mice. More sensitive than [^18^F]THK5117 in PS19 strain. No increase in P301L tau mice	Declercq et al., 2016
		tau deposits	Clinically detect tauopathies in human brain. Increased uptake in rTg4510 mice brain both *in vivo* and *in vitro*	Chiotis et al., 2018
			Increased uptake in 6 month old rTg4510 mice	Su et al., 2020
			Increased brain uptake in P301S mice. Fast brain penetration pleataued in the first minute	McMurray et al., 2021
	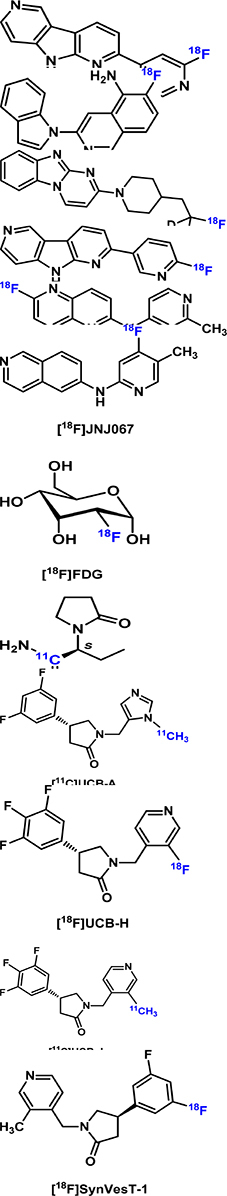		Second-generation Tau tracer with improved specificity. Only tested in human and healthy animals	Chotipanich et al., 2020
				Guehl et al., 2019
				Sanabria Bohórquez et al., 2019
				Wong et al., 2018
				Leuzy et al., 2019
				Baker et al., 2021
Glucose		Glucose metabolism	Brain [^18^F]FDG PET primarily indicates synaptic activity. Hypometabolism correlates well with severity of cognitive deficits. Contradictory results found in diverse AD mouse strains.	Chiaravalloti et al., 2018
SV2A		Synaptic vesicle glycoprotein as a general biomarker of synaptic density	Imaging data unavailable	Cai et al., 2014
			Testing in human showed slow brain kinetics	Zheng et al., 2022
			First tested in human; low specific binding signal in human brain	Bahri et al., 2017
			High specific binding signal in non-human primates and humans. Decreased uptake in APP/PS1 mice, but no difference in tg-ArcSwe mice	Xiong et al., 2021
			Decreased uptake in APP/PS1 and dKI mice	Naganawa et al., 2021
TSPO	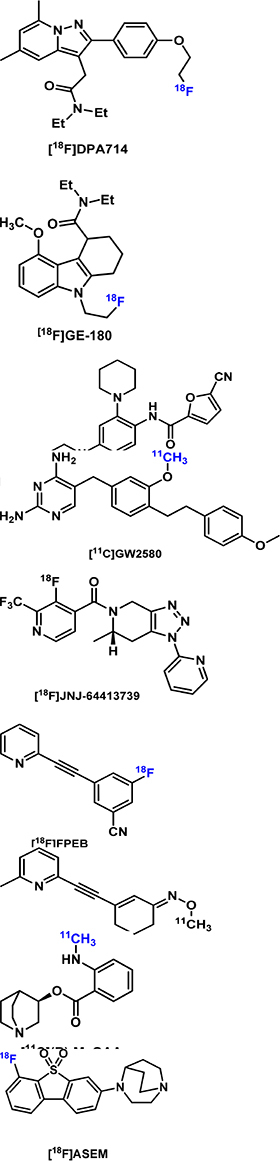	TSPO protein mainly in the outer mitochondrial membrane	Higher [^18^F]DPA714 uptake was noted in the cortex and hippocampus of 12–13 and 15–16-months-old but not younger AD mice compared with control mice	Hu et al., 2020
			May be useful for tracking TSPO/neuroinflammation in early-stage AD but not for monitoring disease progression in APP23 mice	Fan et al., 2016
CSF1R		CSF1R protein on microglia, infiltrating macrophages/monocytes and dendritic cells in the brain	Increased uptake in the brain of AD mice with overexpression of APPswe and APP Indiana mutations	Horti et al., 2019
		CSF1R protein mainly in microglia	Higher sensitivity than [^11^C]CPPC in APP-KI mice	Zhou X. et al., 2021
P2 × 7R		Purinergic P2 × 7 receptor expressed in M1 microglia	Elevated uptake in the LPS-treated site of rat brain compared with contralateral hemisphere	Berdyyeva et al., 2019
mGluR5		Seven-transmembrane G protein-coupled receptors located in excitatory synapses and in glial cells	Contradictory results found in different AD strains	Varlow et al., 2020
			Signal levels correlate with progressive brain atrophy during the aging process in rTg4510 mice	Fang et al., 2017
Cholinergic α7 nAChR		Cholinergic α7 nAChR	Uptake was found increased in aged monkeys	Nakaizumi et al., 2018
			Decreased uptake was seen in aged TgF334 rats	Horti et al., 2014

Currently, PET radiotracers targeting Aβ plaque, tau pathology, synaptic density, and neuroinflammation have been tested on several major AD models for their relatively prominent and consistent pathological phenotypes. To retrieve the most approximate characteristics of the tracer in humans, the proper selection of AD models is key to the success of tracer development, as the neuropathological features vary based on different AD animal models. The selection of a proper AD model is also pivotal to the longitudinal and mechanistic studies of AD and anti-amyloid treatments (Manook et al., 2012; Snellman et al., 2013, 2017). One important point to bear in mind is that when choosing AD animals for PET imaging, correlation with behavioral measures, not just the AD pathologies, should be presented, and the time frame for imaging should also match those for the appearance of the biological phenotypes and related behavioral alterations.

The major advantage of using rat models of AD pathologies is their relatively large brain size, which reduces the partial volume effects in quantitative PET imaging analysis (Toyonaga et al., 2022). Rats are easier to handle than mice, less readily stressed by humans, and produce more robust behavioral testing results (Long and Holtzman, 2019). In addition, the APP/PS1 rats develop tau pathology in the brain, while the APP/PS1 mice with the same promoter lack tau pathology, indicating the APP/PS1 transgene in rats produces closer neuropathology to humans than in mice (Pini et al., 2016).

The translation and clinical Aβ, tau, and FDG PET imaging have transformed our understanding of AD (Scheltens et al., 2021), generated new insights (Aschenbrenner et al., 2018), and opened an avenue for the early detection of AD (Frisoni et al., 2017). With the development of new PET imaging tracers, we expect to gain a deeper understanding of AD at the systemic level and hopefully discover and validate new treatment targets beyond Aβ and tau.

## Author Contributions

BC, ZC, YH, and BM-N contributed to the conception and design of the review. BC wrote the first draft of the manuscript. BC, BM-N, and ZC wrote sections of the manuscript. ZC, BC, and YH revised the manuscript and approved the final version. BC, EB, TT, and JT prepared the figure and table. All authors contributed to manuscript revision, read, and approved the submitted version.

## Author Disclaimer

The contents are solely the responsibility of the authors and do not necessarily represent the official view of the funding agencies.

## Conflict of Interest

The authors declare that the research was conducted in the absence of any commercial or financial relationships that could be construed as a potential conflict of interest.

## Publisher’s Note

All claims expressed in this article are solely those of the authors and do not necessarily represent those of their affiliated organizations, or those of the publisher, the editors and the reviewers. Any product that may be evaluated in this article, or claim that may be made by its manufacturer, is not guaranteed or endorsed by the publisher.
